# Irradiation-induced sterility in an egg parasitoid and possible implications for the use of biological control in insect eradication

**DOI:** 10.1038/s41598-021-91935-4

**Published:** 2021-06-10

**Authors:** Kiran Jonathan Horrocks, Gonzalo Andres Avila, Gregory Ian Holwell, David Maxwell Suckling

**Affiliations:** 1 School of Biological Sciences, Auckland Mail Centre, University of Auckland, Private Bag 92019, Auckland, 1142 New Zealand; 2 The New Zealand Institute for Plant and Food Research Limited, Auckland Mail Centre, Private Bag 92169, Auckland, 1025 New Zealand; 3 The New Zealand Institute for Plant and Food Research Limited, Christchurch Mail Centre, Private Bag 4704, Christchurch, 8140 New Zealand; 4 Better Border Biosecurity, Auckland, New Zealand

**Keywords:** Agroecology, Behavioural ecology, Invasive species, Environmental impact, Animal behaviour, Entomology

## Abstract

Classical biological control is a pest control tool involving the release of imported natural enemies. The Sterile Insect Technique (SIT) comprises releasing sexually sterile insects of a pest into the wild population for suppression or eradication. Both these approaches are environmentally friendly and their combination can result in a synergistic impact on pest populations and improve eradication. However, stringent regulation surrounding the introduction of biological control agents limits their use in eradication owing to the perceived risk of effects on non-target organisms. We investigated the irradiation biology of the egg parasitoid *Trissolcus basalis* to ascertain whether sterile parasitoids could mitigate the risk of potential sustained non-target impacts. Mated female *T. basalis* were gamma-irradiated at doses between 120 and 150 Gy and exposed to egg masses of their host *Nezara viridula* throughout their lifespans. This resulted in host mortality, despite a substantial reduction in developing parasitoid offspring, which followed a negative dose–response. There was no emergence of parasitoid offspring at 140 Gy and above. Irradiation did not affect oviposition behaviour but caused an increase in longevity. Consequently, sterile parasitoids could possibly alleviate concerns regarding the irreversibility of biological control release, which promotes further investigation of their potential role in eradication.

## Introduction

Classical biological control (CBC) involves the introduction of natural enemies to reduce the impact of target pests^1^, and is often a fundamental component of integrated pest management (IPM) programmes^2,3^. Unlike pesticides, natural enemies are self-sustaining, generally environmentally benign, and do not cause target pest resistance^4,5^. Despite the rarity of population-level impacts on non-target organisms, it remains a concern when considering the introduction of a CBC agent^4^. Consequently, some governments impose risk-attentive regulations to assess potential risk prior to the release of new CBC agents, which increases the expense of biological control release applications and limits their approval^6–8^.

Another effective and benign approach—the Sterile Insect Technique (SIT)—involves mass-rearing and repeatedly releasing sexually sterile conspecifics into the target pest population, at a ratio higher than the wild population. Generally, males are sterilised by gamma or X-ray radiation and released to mate with wild females, which inhibits offspring production and causes a reduction in the F_1_ generation^9^. The SIT is primarily utilised as a component of area-wide IPM, which involves managing a pest population within a defined area^10^, but is also used in eradication programmes^9,11^. If practicable, eradication is likely to be more beneficial than long-term management because it averts the accumulation of both ecological and economic impacts^12,13,14^. The SIT also poses virtually no non-target or environmental risks, and has increased efficacy at lower pest densities^15^.

The combination of biological control with SIT was first theorised by Knipling^15^ and Barclay^16^, who found that both techniques should interact synergistically, with each method exerting a greater impact on the target pest than if it were used alone. However, given the stringent regulations surrounding biosafety assessment of CBC agents and importation in many countries, and consequent length of time from application to approval^6^, it would be difficult to safely introduce an agent in time to be utilised in an eradication response^13^. The possibility of the CBC agent persisting on non-target organisms post-eradication would also restrict the implementation of biological control in such a programme.

These regulatory issues concerned with the irreversibility of CBC could potentially be mitigated by also applying SIT to a candidate CBC agent, such as parasitoids. Previous research has shown that irradiating female parasitoids can induce sterility, which does not affect their ability to kill the host, but the purpose of these studies was to investigate host immune response^18–20^. Theoretically, released sterile female parasitoids could therefore kill the target pest without producing viable offspring for their population to persist. However, there is evidence that inundative parasitoid releases may sometimes exert population-level non-target effects, though the impact is predictably local and transient^4,21^. Consequently, the release of sterile parasitoids as part of an eradication program may not be completely devoid of risk^22^. This novel approach has been termed the Kamikaze Wasp Technique (KWT), which was proposed recently as a potential synergistic component of insect eradication^23^.

The irradiation biology of the egg parasitoid *Trissolcus basalis* Wollaston (Hymenoptera: Scelionidae) was investigated by undertaking dose–response experiments to ascertain whether sterility could be induced, and whether this would affect the wasps’ ability to impact the survival and development of its host *Nezara viridula* Linnaeus (Hemiptera: Pentatomidae). Post-irradiation behaviour and fitness were also considered. This represents the first step required to examine the feasibility of the KWT. However, this constitutes a model system that lends support to the ongoing SIT work on *Halyomorpha halys* Stål (Hemiptera: Pentatomidae) as a potential means to eradicate this emerging pest^24,25^, as its parasitoid *Trissolcus japonicus* (congener to *T. basalis*) could similarly be employed for use in the KWT. Consequently, *T. basalis* is not being proposed as a candidate for the KWT but it serves as a model.

## Methods

### Source of insects

The *N. viridula* hosts and *T. basalis* parasitoids used for experiments were supplied from established colonies that were maintained in a controlled temperature room (25 ± 1 °C, 16:8 h L:D) at The New Zealand Institute for Plant and Food Research Limited, Auckland, New Zealand. The *N. viridula* colony was established from adults collected from tomato plants in Ruawai (36°06′06″ S, 174°00′05″ E) and from *Cleome* sp. at Kelmarna Gardens, Auckland (36°51′03.3″ S, 174°43′58.7″ E). The *T. basalis* colony was established from parasitised *N. viridula* egg masses (eggs turning black) collected from a number of host plants at Kelmarna Gardens. Host plants included beans, tomatoes^26^, and *Cleome* sp.^27^. The use of plants for insect collection complied with institutional, national and international guidelines.

### Insect rearing

#### N. viridula

Egg masses were collected from the adult portion of the *N. viridula* colony and kept in 9 cm Petri dishes. When these eggs were visibly close to hatching, the emerging nymphs were provided with moist cotton wool as a water source. When these moulted into second instar nymphs, green beans were provided as a food source. Upon reaching the third instar, the nymphs were transferred to a plastic rearing cage (69 cm diameter × 19 cm height) containing filter paper on the bottom surface, on which moist cotton wool and green beans were provided. When nymphs reached the fourth instar, the conditions in the rearing cage remained identical except for the introduction of raw peanuts as a protein source. Fifth instar nymphs that had moulted into adults were then transferred into adult rearing cages (69 cm diameter × 20 cm height). Wax paper, folded into a fan, was introduced into each of these cages as an oviposition surface. Food, water, and filter paper were changed twice weekly for all rearing cages.

#### Trissolcus basalis

Parasitoids were reared within clear plastic vials (8.5 cm diameter × 6 cm height) inside a nylon mesh cage (69 cm × 48 cm × 48 cm), in the same location and under the same conditions as the *N. viridula* colony. A portion of the host egg masses from the *N. viridula* colony was collected daily to maintain the *T. basalis* colony. These were stored at 10 °C for up to a week if not immediately exposed to *T. basalis*, after which they were transferred to a freezer (− 20 °C). Frozen *N. viridula* egg masses were used to maintain the colony if fresh masses were not available at the time^28,29^. Upon emergence from the initial parasitised *N. viridula* egg masses, at least two days were given for females to mate. Every second to fourth day, three to five mated females, no older than 10 days, were removed into individual vials and each exposed to a single *N. viridula* egg mass (40–100 eggs) for 48 h. A drop of honey (~ 10 µL) was provided as a food source in every vial, which was replenished (~ 100 µL) upon the emergence of *T. basalis* offspring.

### Irradiation dosimetry trials

#### Irradiation

Our methodology was adapted from that of Soller and Lanzrein^18^ and Tillinger et al.^19^, who sterilised female parasitoids to examine host immune response in the absence of developing parasitoid offspring. Male *T. basalis*, which eclose first^30^, were separated from the rearing vials before females began to emerge. A single female was then introduced into a vial containing a separated male, and left until mating was observed.

Five mated female *T. basalis* between 2 and 4 days old were placed into each of five vials (4 cm diameter × 5 cm height), which were then plugged with cotton. Four of the vials were then covered within a thick-walled plastic box, as cobalt^60^ radiation interacts with matter through the Compton effect, whereby electronic equilibrium of photons is not reached until a few millimetres of penetration^31^. The box therefore ensures that the set radiation dose is fulfilled before the beam reaches the insects. The vials were placed within the irradiation field whilst lowering the irradiator (Eldorado 78, Foss Therapy Services, North Hollywood) to give the maximum possible dose rate of 2.4 Gy/min. Vials were laid on their side to prevent inconsistency in received dosimetry through vertical movement of parasitoids under the radiation source. One vial of female parasitoids was removed when exposure reached each of 120 Gy, 130 Gy, 140 Gy and 150 Gy, which was based on timing. This was replicated twice, giving a total of 10 female *T. basalis* for each irradiation dose tested and for the non-irradiated positive control.

Upon completion of irradiation, parasitoids were returned to the laboratory (25 ± 1 °C, 16:8 h L:D) where irradiated females were individually separated into vials (8.5 cm diameter × 6 cm height), noting dosage, and rested for 24 h with a drop of honey (~ 10 µL). When possible, a single fresh *N. viridula* egg mass, no older than 24 h^32^, was placed into each vial and oviposition allowed for 24 h. However, to ensure an adequate supply, fresh *N. viridula* egg masses were stored at 10 °C for no longer than a week to halt their development prior to exposure to irradiated *T. basalis* females^33,34^. To ascertain whether induced sterility is permanent, these females were exposed to a new egg mass weekly until death. Any parasitoids that did not oviposit within 20 min were given a new egg mass, and re-observed. The mean number of eggs in masses exposed to parasitoids were 53, 51, 47, 47 and 54 for 120 Gy, 130 Gy, 140 Gy, 150 Gy and the non-irradiated controls, respectively.

#### Assessment of sterility

To evaluate parasitism for each host egg, nymphal development of *N. viridula* was assessed. This was necessary because it was possible that oviposition by irradiated females would not produce a developing parasitoid offspring (will not turn black), instead with host death occurring during oviposition, leaving no sign of either parasitoid or host development^18,35^. Eggs that became pink were recorded as containing a developing *N. viridula* nymph^36^. Any infertile eggs, which retained a pale colour with no change^37^, were not counted as parasitised, as it was not possible to determine the cause of the lack of *N. viridula* nymph development. Host eggs that did not either retain a pale colour or contain a developing host, or where a parasitoid offspring developed, were therefore recorded as parasitised, which also represents host death. Where these indicators of parasitism were seen without its occurrence during the observation period, oviposition was retrospectively confirmed and recorded as above.

The proportion of parasitised *N. viridula* eggs with developing parasitoids (i.e. eggs turn black), emerged parasitoids, and the sex ratio of any emerged offspring, were recorded for each parasitised egg mass to investigate the degree of induced sterility. Any egg containing a developing parasitoid that did not emerge within three weeks of oviposition by an irradiated mother was dissected to confirm its presence, which was recorded. The sex, level of development, and living status were also recorded. Eggs in which it was difficult to decipher whether a parasitoid or host had developed were also dissected to ensure that the result was recorded correctly. Any *N. viridula* offspring that successfully emerged was also recorded. Percentage F_1_ development and emergence were recorded as the proportion of offspring that either developed or emerged from eggs in which an irradiated *T. basalis* oviposited. This experiment was repeated with non-irradiated female *T. basalis* as a positive control. As a negative control, we left a fresh *N. viridula* egg mass to develop inside a vial without the presence of a parasitoid. This was replicated 21 times to ascertain natural host development rates and to confirm that host death was due to parasitism by irradiated *T. basalis*. To replicate the conditions that egg masses were subjected to before parasitoid exposure, they were collected at no older than 24 h and accumulated at 10 °C for up to a week. The time that each egg mass spent at 10 °C was also recorded.

#### Post-irradiation fitness and behaviour

Time spent searching for the host, drumming antennae on host eggs, ovipositing in first egg, and time taken to initiate the first oviposition were recorded for *T. basalis* females during each host exposure to ascertain potential impacts of irradiation on oviposition behaviour^32^. For drumming, recording of time continued if the parasitoid temporarily left the egg mass after locating it. The total number of host eggs attacked and longevity were recorded for *T. basalis* females as indicators of post-irradiation fitness^20,38^.

### Statistical analysis

Binomial generalised linear models (GLM) (logit link) were used to assess the relationship between the irradiation dose for *T. basalis* females and both percentage F_1_ development and percentage F_1_ emergence. Binomial GLM’s (logit link) were also used to assess the potential difference in offspring sex ratio between irradiated and non-irradiated parasitoid mothers, as well as for the negative control. Infertile eggs were excluded from the analysis of the negative control data, as they were not considered for the confirmation of parasitism by irradiated parasitoids. Poisson GLM’s (log link) were used to assess the effect of irradiation dose on the lifetime sum of host eggs attacked and the change in the mean number of eggs parasitised over time, for each irradiation dose individually. Poisson GLM’s (log link) were also used to explore the relationship between mean longevity and irradiation dose, and for each component of the oviposition sequence compared with dose. Binomial (logit link) and Poisson (log link) GLM’s, for proportion and count data respectively, were used where comparisons were made between irradiated and non-irradiated parasitoids as distinct groups. Analysis of variance (ANOVA) was applied on all GLM’s. Statistics presented were generated in R (version 3.6.0; R Foundation for Statistical Computing, Australia) using the packages ggplot 2^39^ for plots and lme4^40^ for analyses.

## Results

### Irradiation dosimetry

**Figure 1:**
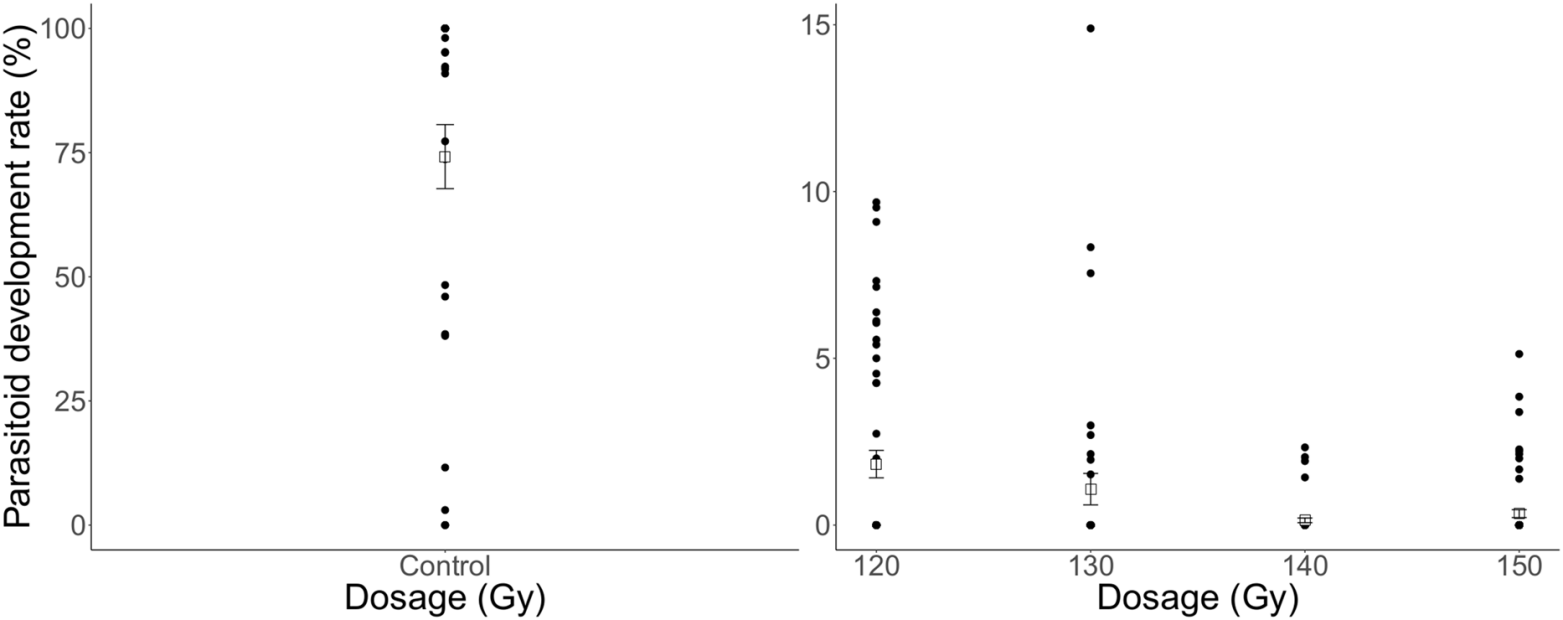
Lifetime percentage of *Nezara viridula* eggs attacked by irradiated and non-irradiated female *Trissolcus basalis*, which resulted in developed offspring of the latter. Squares and error bars represent means and standard error, respectively.

The percentage of host eggs attacked throughout the lifetime of *T. basalis* females, which resulted in developing *T. basalis* offspring, decreased with an increasing irradiation dose (F = 13.39, df = 3, P < 0.001) (Fig. 1). The means for 120 Gy, 130 Gy, 140 Gy, and 150 Gy were 1.83%, 1.08%, 0.14%, and 0.34%, respectively. However, there was considerable variation, most notably at 130 Gy where host egg exposures resulted in between 0 and 15% development of offspring. Conversely, the non-irradiated *T. basalis* females produced a substantially higher percentage of developing offspring, with a mean of 74.19% (F = 1528.80, df = 1, P < 0.001). However, there was also high variability in the non-irradiated control, which ranged between 0 and 100% development of offspring.

**Figure 2:**
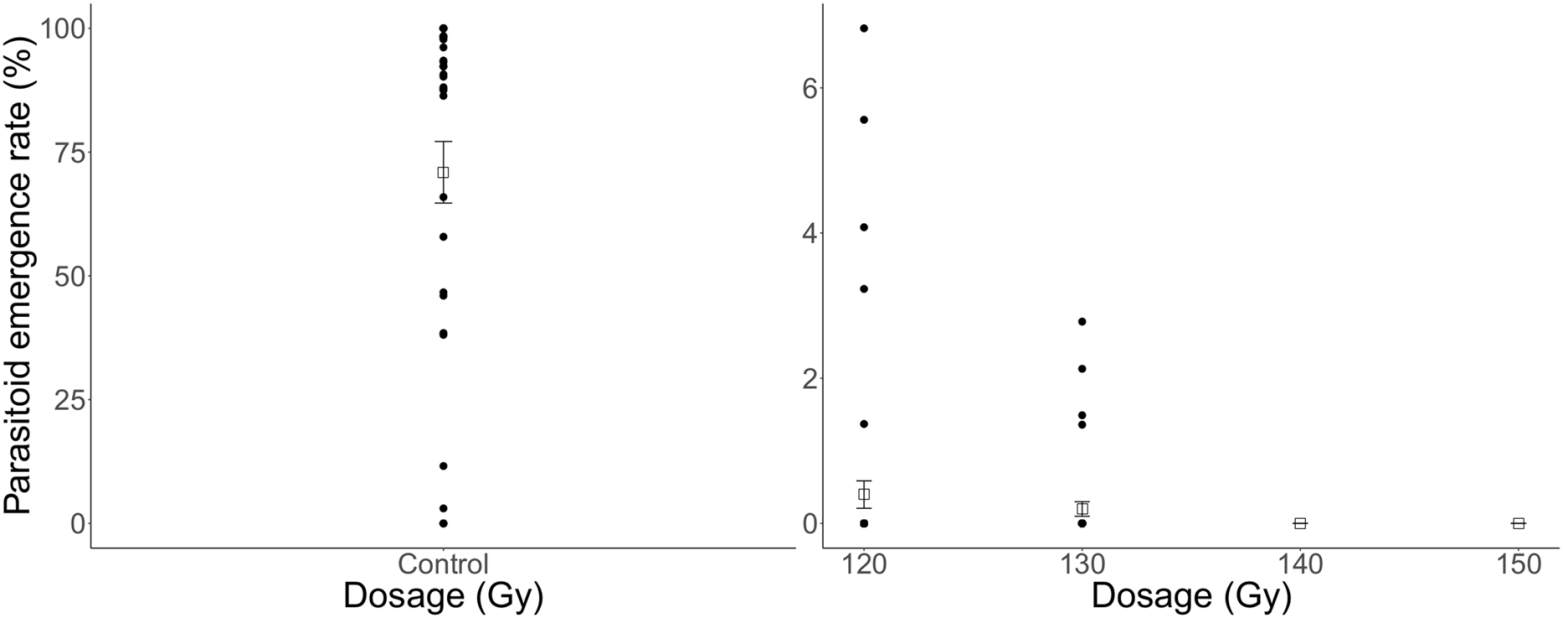
Lifetime percentage of *Nezara viridula* eggs attacked by irradiated and non-irradiated female *Trissolcus basalis*, which resulted in emerged offspring of the latter. Squares and error bars represent means and standard error, respectively.

The percentage of eggs parasitised by irradiated female *T. basalis* that resulted in emerging offspring showed a similar relationship (F = 14.65, df = 3, P < 0.001) (Fig. 2). The means for 120 Gy and 130 Gy were 0.4% and 0.2% emergence, respectively, whereas 140 Gy and 150 Gy produced no successfully emerging offspring. Higher variability was observed at 120 Gy, with 6.82% emergence resulting from one host egg exposure. There was a significantly higher percentage of emerging offspring in the non-irradiated group, with a mean of 70.92% (F = 1976.20, df = 1, P < 0.001). There was also high variability in the non-irradiated control.

Of the non-emerged progeny from sterile female *T. basalis* irradiated at 140 Gy, all five were female, with two of these only partially developed. Amongst the offspring of sterile females irradiated at 150 Gy, that did not emerge, were seven females and one male, and three of unknown sex, with eight of these only partially developed. Of both the emerged and non-emerged progeny from the non-irradiated *T. basalis* females, 962 were female and 171 were male, all of which were fully developed. The sex ratio of developed offspring from sterile *T. basalis* mothers was similar to that for non-irradiated mothers (F = 0.05, df = 1, P = 0.827).

The negative control demonstrated that *N. viridula* egg masses stored at 10 °C for between one and seven days expressed an average of 96% development of *N. viridula* offspring (Table 1). There was no significant difference in the percentage of developing offspring between egg masses that had been stored at 10 °C for different time periods (F = 0.56, df = 6, P = 0.755).

**Table 1:** Negative control showing the percentage of eggs that developed *Nezara viridula* nymphs after egg masses were kept at 10 °C for up to a week (N = 3 egg masses per day spent in refrigerator; 21 egg masses in total).

Days in fridge	Average percentage development
1	97.74
2	91.16
3	99.24
4	94.95
5	97.47
6	98.67
7	90.30
Overall mean	96.00
Overall assessment	F = 0.56 P = 0.755

### Post-irradiation fitness and behaviour

Oviposition behaviour was similar across all irradiation doses and the non-irradiated controls, with no discernible relationships between dose and mean time spent undertaking each step in the oviposition sequence (searching: F = 0.46, df = 4, P = 0.766; antennal drumming: F = 1.91, df = 4, P = 0.109; time taken to oviposit: F = 1.20, df = 4, P = 0.310; time spent ovipositing: F = 1.12, df = 4, P = 0.354) (Table 2). The irradiated group was also similar to the control group for each step in the oviposition sequence (searching: F = 1.28, df = 1, P = 0.260; antennal drumming: F = 0.01, df = 1, P = 0.924; time taken to oviposit: F = 0.20, df = 1, P = 0.652; time spent ovipositing: F = 0.10, df = 1, P = 0.755) (Table 2). High standard deviations demonstrate considerable variance in the time taken to complete each step in the oviposition sequence for both irradiated and control *T. basalis* females.

**Table 2:** Mean time spent (seconds), by irradiated and control female *Trissolcus basalis* throughout their lifespans, undertaking each step in the oviposition sequence.

Irradiation dose (Gy)	Step in oviposition sequence							
	Searching	SD	Antennal drumming	SD	Time to first oviposition	SD	Ovipositing in first egg	SD
120	92.88	68.05	171.46	165.08	264.54	165.03	243.37	113.17
130	94.47	51.64	189.14	257.43	283.50	277.72	260.34	139.99
140	99.16	101.81	107.10	83.67	210.18	133.51	212.24	114.83
150	90.18	71.52	143.94	153.48	234.12	165.02	223.65	109.71
Control	111.06	106.22	146.56	100.20	259.94	149.45	239.47	125.41
Overall assessment	F = 0.46 P = 0.766		F = 1.91 P = 0.109		F = 1.20 P = 0.310		F = 1.12 P = 0.354	
Irradiated vs control	F = 1.28 P = 0.260		F = 0.01 P = 0.924		F = 0.20 P = 0.652		F = 0.10 P = 0.755	

**Figure 3:**
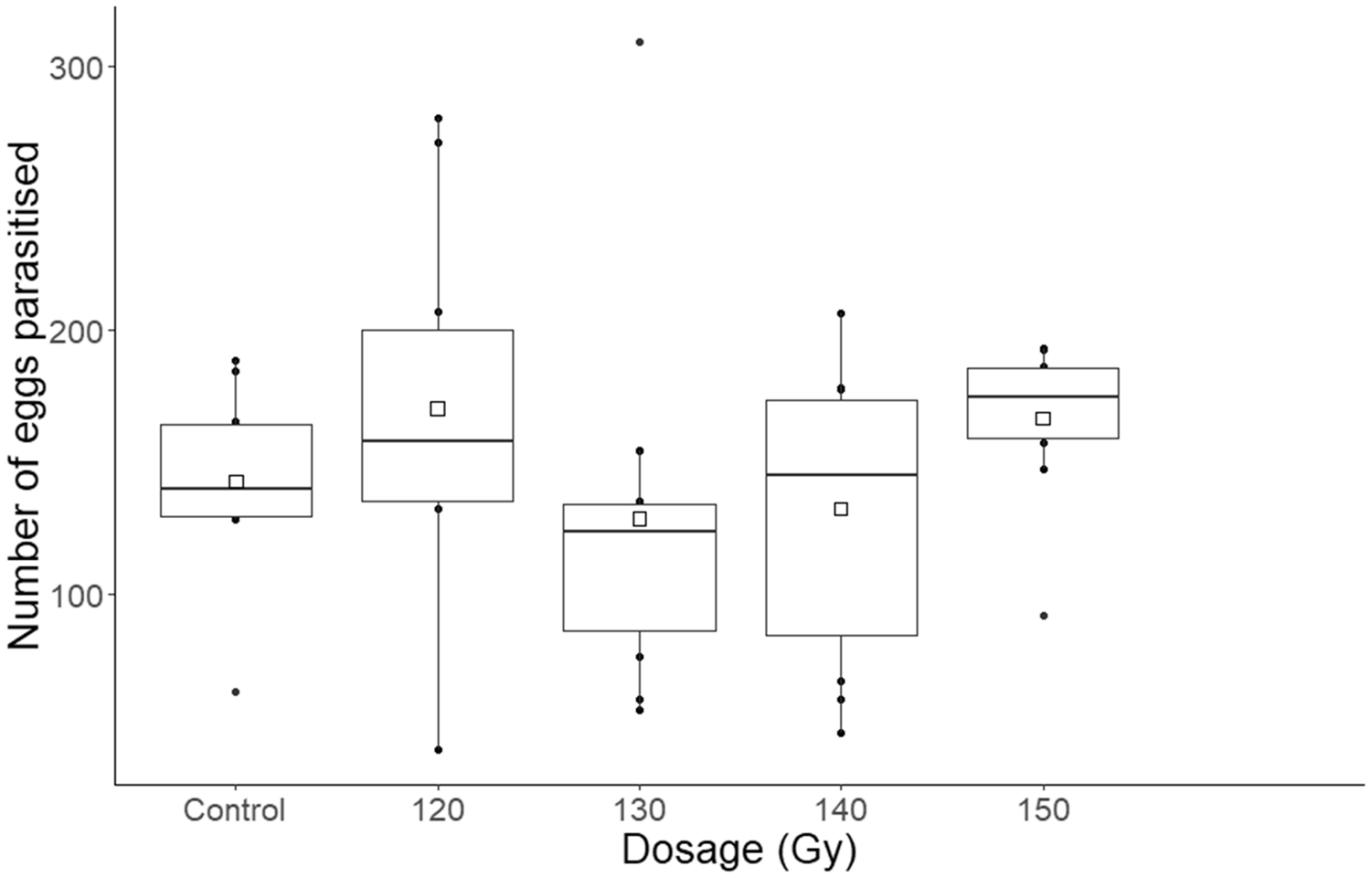
Boxplots of the total number of *Nezara viridula* eggs parasitised by non-irradiated female *Trissolcus basalis*, and by those subjected to increasing irradiation doses, throughout their lifespans. The box represents the interquartile range, and the whiskers are 1.5 times the interquartile range. Squares represent means.

**Figure 4:**
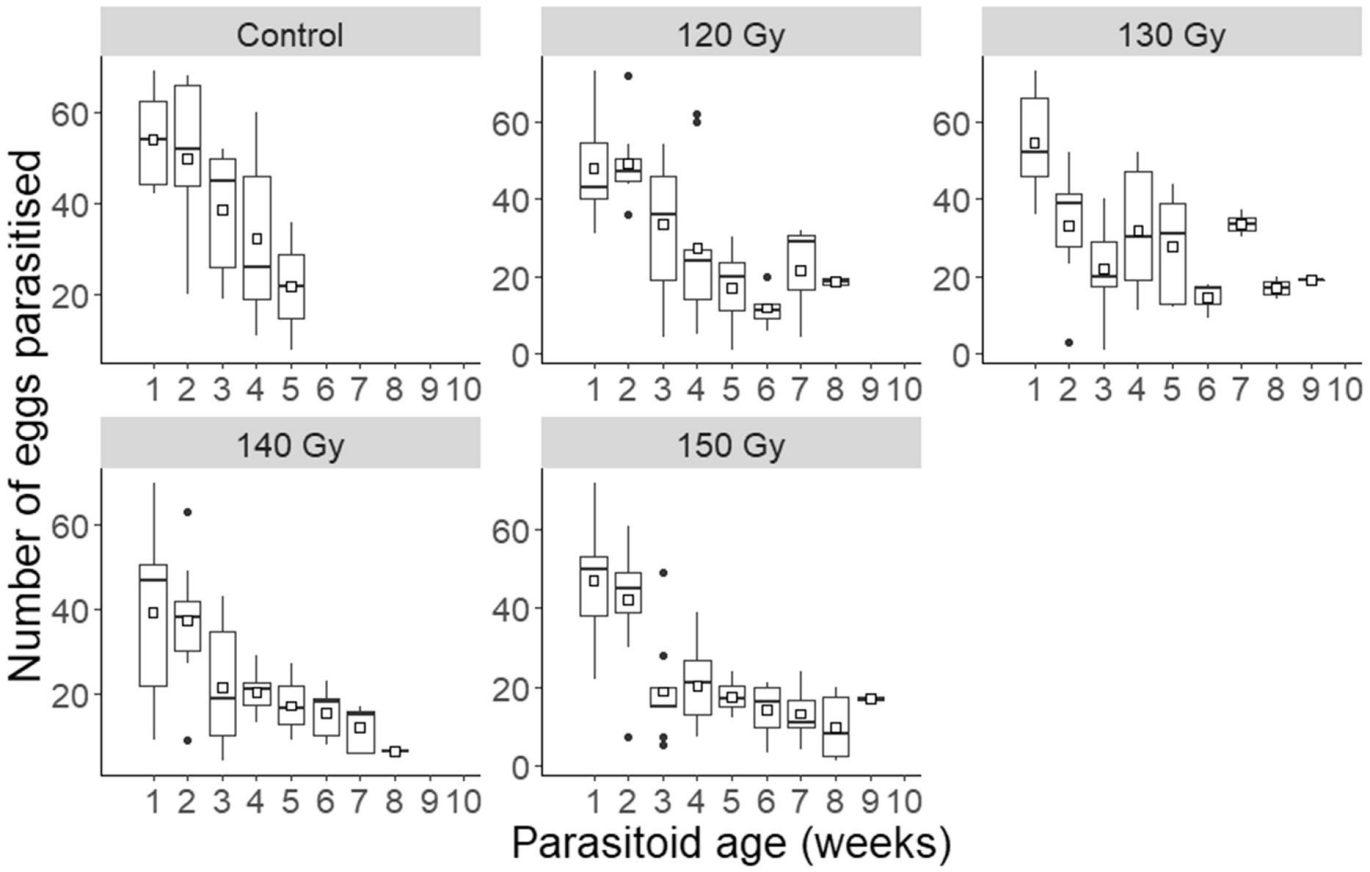
Boxplots of the number of *Nezara viridula* eggs parasitised by non-irradiated female *Trissolcus basalis*, and by those irradiated between 120–150 Gy, during each weekly host egg exposure. The box represents the interquartile range, and the whiskers are 1.5 times the interquartile range. Squares represent means.

The total number of eggs parasitised and killed by *T. basalis* females over their lifespans was not related to irradiation dose (F = 1.17, df = 4, P = 0.337) (Fig. 3). Similarly, the lifetime number of eggs parasitised and killed did not vary strongly between irradiated and non-irradiated females (F = 0.11, df = 1, P = 0.742). Females irradiated at 120 Gy exhibited the highest mean number of eggs parasitised, at 170.10, whilst those irradiated at 130 Gy expressed the lowest, at 128.40. Irradiated parasitoids exhibited an abrupt decrease in the number of eggs parasitised at either the second or the third week of host egg exposure (Fig. 4). This decrease continued incrementally until either the eighth or the ninth week of exposure. Conversely, the non-irradiated *T. basalis* females demonstrated higher parasitism each week, but over a shorter period, with no oviposition occurring after week five. This negative relationship between parasitoid age and number of host eggs parasitised was statistically significant for all irradiation doses and the positive control (120 Gy: F = 6.47, df = 7, P < 0.001; 130 Gy: F = 3.85, df = 8, P = 0.003; 140 Gy: F = 6.55, df = 7, P < 0.001; 150 Gy: F = 11.31, df = 8, P < 0.001; control: F = 3.25, df = 4, P = 0.027). Irradiated parasitoids therefore parasitised and killed a similar number of hosts throughout their lifespans compared with non-irradiated parasitoids (Fig. 3), but over a longer period (Fig. 4).

**Figure 5:**
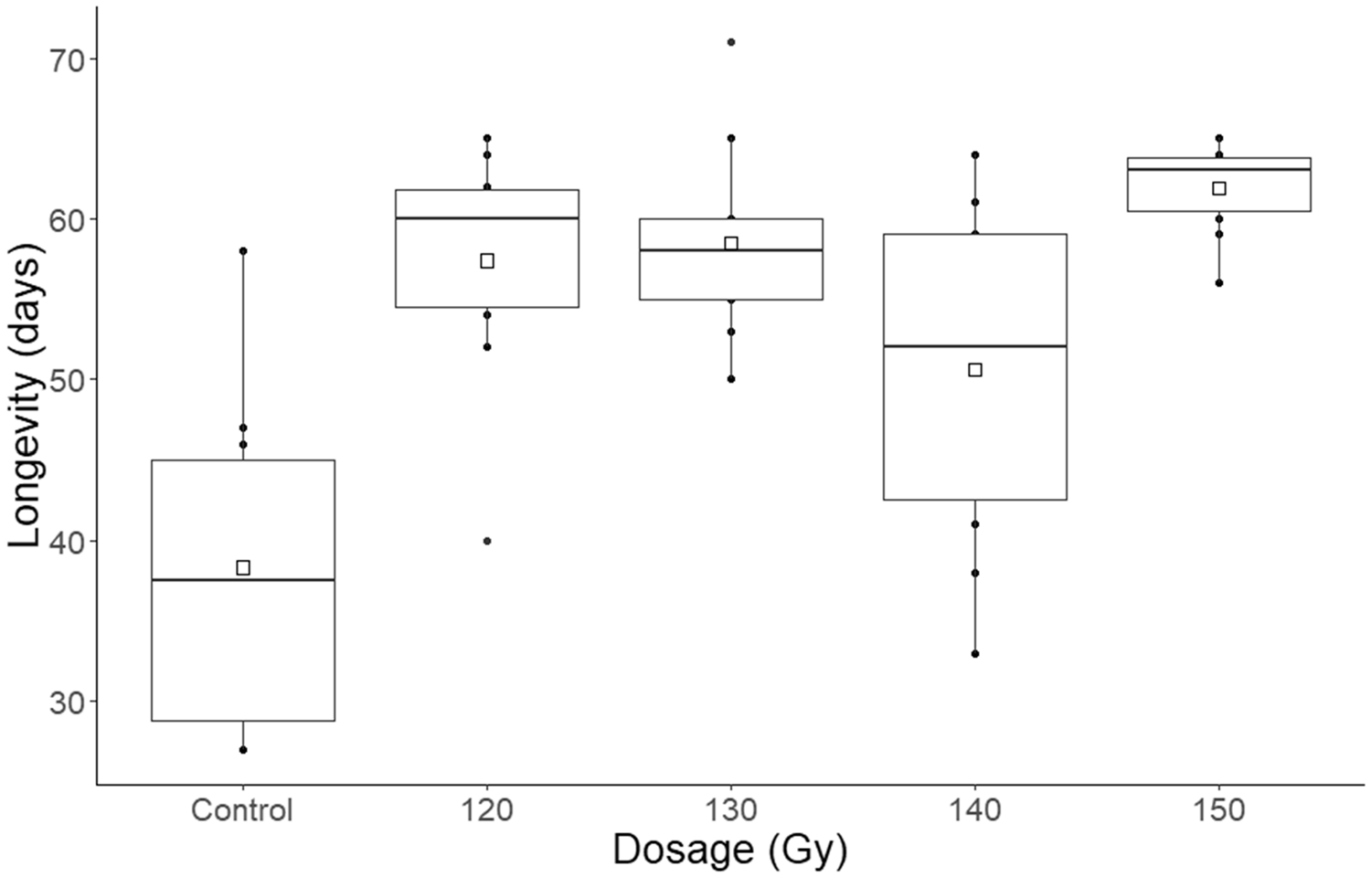
Boxplots of the longevity of irradiated and non-irradiated *Trissolcus basalis*. The box represents the interquartile range, and the whiskers are 1.5 times the interquartile range. Squares represent means.

There was no discernible relationship between irradiation dose and mean longevity amongst the irradiated *T. basalis* females, where the lowest was 50.6 days at 140 Gy and the highest was 61.9 days at 150 Gy (F = 0.23, df = 1, P = 0.632) (Fig. 5). However, longevity for the non-irradiated group was substantially shorter than for the irradiated group with a mean of 38.30 days (F = 39.48, df = 1, P < 0.001).

## Discussion

### *Sterility of* T. basalis *and the potential of the KWT*

A substantial dose-dependent reduction in the proportion of developing offspring from irradiated female *T. basalis* was observed. This sterility is likely to have been caused by damage to the ovaries or oocytes, inhibiting the production of viable eggs^41^. Grosch and Sullivan^42^ confirmed this as the cause of irradiation-induced sterility in female *Bracon hebetor* Say (Hymenoptera: Braconidae). This is corroborated by more recent studies on irradiation-induced sterility in *Chelonus inanitus* Linnaeus (Hymenoptera: Braconidae)^18^ and *Glyptapanteles liparidis* Bouche (Hymenoptera: Braconidae)^19^, which resulted in the deposition of inviable eggs. These studies used irradiated parasitoids to assess host immune response in the absence of developing parasitoid offspring, rather than for the potential use of sterile parasitoids as part of a biosecurity response. However, Grosch and Sullivan^42^ noted that females successfully oviposited viable eggs produced prior to irradiation. This could explain the small number of female *T. basalis* amongst the developing F_1_ progeny from sterile females, indicating that some of the eggs produced before irradiation were successfully fertilised. It could also explain the lack of visual evidence for sterile *T. basalis* eggs inside parasitised host eggs, which was therefore an unsuitable method for confirming oviposition.

The presence of only one male amongst the developing offspring from sterile *T. basalis* mothers is unsurprising given the substantially higher ratio of female offspring in the non-irradiated control, which occurs in many hymenopteran parasitoids. This is due to a mating system with strong local mate competition where the mother produces only enough sons to mate with all of her daughters^43^.

The ability of irradiated *T. basalis* to kill host eggs, despite the absence of developing offspring, is supported by the findings of Soller and Lanzrein^18^, and Tillinger et al.^19^, who observed that irradiation does not affect the production and injection of venom and parasitoid-associated factors by parasitoid females. Irradiated females of the egg parasitoid *Trichogramma evanescens* Westwood (Hymenoptera: Trichogrammatidae) also retain their ability to kill the host^20^. Scelionid parasitoids do not possess a venom gland^35,44^, but all non-venomous egg parasitoids kill their hosts during oviposition, even when offspring fails to develop, through mechanisms such as mechanical damage from ovipositor insertion, or the injection of growth arrestment factors^44,45^. Irradiated *T. basalis* females therefore retained their ability to kill the host. Furthermore, the negative control resulted in a high percentage of host development for *N. viridula* egg masses in the absence of a *T. basalis* female, indicating that parasitism by sterile parasitoids resulted in host death. This also validates a lack of host development as the methodological indicator for confirming parasitism.

Complete sterility was achieved with no F_1_ emergence from *T. basalis* females irradiated at 140 Gy and above. These results suggest that in a hypothetical field release, sterile *T. basalis* females could kill the target host, but it would not persist in the environment to potentially exert sustained non-target impacts after the pest is eradicated^4,46^. It is therefore possible that sterile ‘kamikaze wasps’ could be utilised synergistically in an eradication programme alongside SIT, or other tactics, to mitigate regulation associated with the perceived irreversible risk of exotic parasitoid introduction^6–8^. However, testing whether higher irradiation doses could eliminate any development of female offspring would be beneficial from a biosafety perspective, but may lead to reduced fitness^19,47,48^.

Regardless, the possible risk of ephemeral, but non-negligible, non-target impacts from inundatively released sterile parasitoids must be considered^4,21^. Well-known determinants of successful eradication include early detection of invasion and rapid response, with elimination generally viewed as impracticable if a pest has spread widely over a variety of habitats^49–51^. Eradication responses therefore tend to be highly targeted to specific urban locations, where incursions often begin^49,52^. This alleviates the concern of transient environmental risk from sterile parasitoids as they would not be released in large numbers across an array of non-target habitats. However, due to the complicated set of scientific, economic, and socio-political requirements, much of the scientific community questions the feasibility of eradication compared to long term area-wide management^50,53^.

### Post-irradiation fitness and behaviour

To be effective in SIT programmes, released sterile individuals must retain fitness and normal reproductive behaviour^54^. The oviposition behaviour data suggest that the irradiation doses employed had no impact on any component of the *T. basalis* oviposition sequence. This is the first experimental assessment of the potential impact of irradiation on the oviposition behaviour of an egg parasitoid. However, Soller and Lanzrein^18^ mentioned that X-ray irradiation did not affect the oviposition behaviour of *C. inanitus* (without supporting data), which also attacks the eggs of its host^55^. For species from other insect orders, it is common for irradiated females to deposit substantially fewer eggs than control females, indicating a negative impact on oviposition behaviour^56–58^. Our results indicate that irradiation-induced sterility may not negatively influence oviposition behaviour in certain parasitoids. In the field, sterile wasps would need to retain the behaviours required for final host acceptance and oviposition in order to kill the host. This is analogous to the importance of sterile males retaining their competitive mating ability amongst the wild male population in a conventional SIT scenario^54^.

Fecundity of sterile individuals is a crucial measure of competitive fitness in SIT programmes^54^, as well as an indicator of parasitoid fitness^59,60^. For analysis, we used the number of host eggs parasitised, as opposed to percentage parasitism, because there was slight variability in the number of *N. viridula* eggs in each mass exposed to *T. basalis* females. Irradiation dose did not influence average lifetime parasitism in *T. basalis* females, suggesting that irradiation does not affect fecundity as defined by the number of host eggs attacked and killed. This also demonstrates that irradiation did not influence the lifetime number of hosts killed. Irradiated *C. inanitus* females also do not experience a reduction in oviposition behaviour^18^. However, Tillinger et al.^19^ found that *G. liparidis* irradiated at 48 Gy and 96 Gy exhibited a four-fold reduction in the number of deposited eggs compared with the non-irradiated control, which indicated a significant fitness impact, though the proportion of parasitised hosts that died was similar between these groups. The number of eggs laid by irradiated *B. hebetor* also decreased with an increasing dose^42^, but the number of oviposition attempts was not reported. It is therefore likely that the effect of irradiation on parasitism would vary amongst parasitoid species. Most species in other insect orders commonly targeted by SIT show a decrease in fecundity when irradiated^47,48,58,61,62^. Our fecundity results demonstrate the importance of investigating the potential impact of irradiation on parasitism when assessing the feasibility of the KWT scenario in relation to parasitoid fitness.

Despite this, irradiation clearly affected the decline in the number of host eggs parasitised and killed by *T. basalis* females during each weekly exposure. Irradiated females attacked fewer host eggs with each exposure but continued for a longer period than the non-irradiated controls. This is dissimilar to the effect of irradiation on *G. liparidis*, where non-irradiated females continued to oviposit for twice the amount of time as irradiated females^19^. Similar studies on species from different orders express variation in their findings. For example, Ali et al.^48^ found that female *Mythimna separata* Walker (Lepidoptera: Noctuidae) expressed a higher oviposition rate with an increase in irradiation dose, whereas other studies have observed the opposite effect^47,63,64^. The pattern observed in *T. basalis* is likely due to irradiation damage to the ovaries and/or oocytes, affecting egg production^42,65^, with Tillinger et al.^19^ suggesting this as the cause for a lower oviposition rate in irradiated *G. liparidis* females. This may constitute a negative impact of irradiation on female *T. basalis* fitness, and in the context of the KWT could affect their patch (i.e. single host egg mass) exploitation ability by limiting the number of hosts killed within each visited egg mass^66^. Female *T. basalis* exert more effort defending a patch from super-parasitism when they have laid more eggs^66^. However, parasitising a similar number of eggs over a longer period could be beneficial by improving parasitoid-host synchrony, which becomes an issue when parasitism does not temporally correspond with host availability/susceptibility^67,68^.

Observing a significantly longer lifespan for irradiated *T. basalis* was unexpected, given that it is generally reported that higher doses of irradiation decrease longevity in insects^69^. However, Tillinger et al.^19^ demonstrated that irradiation had no significant impact on the longevity of *G. liparidis* females. Several studies on insect pest orders have also found that irradiation-induced sterility stimulates either an increase in longevity, or has no significant impact^70–72^. In this case, a longer lifespan for irradiated female *T. basalis* was probably due to the lower number of host eggs parasitised per week, with this trade-off between fecundity and longevity being well studied in insects^73–75^. This occurs because an increase in gamete production is costly, and causes a compensatory decrease in longevity^76^. This is also known to occur in parasitoid wasps^77,78^. For example, females of the egg parasitoid *Gryon pennsylvanicum* Ashmead (Hymenoptera: Scelionidae) exhibit a reduction in fecundity when host-deprived, which in turn causes an increase in longevity^77^. For irradiated *T. basalis* females, this does not necessarily indicate a fitness gain, but rather an artificially induced trade-off in fitness between fecundity and longevity^76^. However, it could be beneficial for the KWT in terms of parasitoid-host synchrony^67^, though field trials would be required to assess this.

### Potential application of KWT

The combination of biological control with SIT is well-researched for the suppression or eradication of insect pests, although the integration of biological control with pesticides, host plant resistance, and cultural techniques has also received attention^13,79^. This combination received early theoretical and modelling-based interest from Knipling^16^ and Barclay^17^, who demonstrated that it should have a synergistic impact on a target pest. This is because parasitoids generally target a different host life-stage than does SIT, which targets adults^13^. For example, sterile pest release would decrease the number of fertile eggs produced in the subsequent generation, resulting in a higher ratio of egg parasitoids to hosts and increasing the impact of the parasitoid on the pest. Similarly, the effect of the parasitoid population would result in a higher ratio of sterile to wild pests, increasing the impact of the SIT component^80^.

A few programmes have successfully combined biological control and SIT for pest eradication or suppression. A large-scale example is the eradication of *Anastrepha* spp. Schiner (Diptera: Tephritidae) fruit flies from the Mexican states of Chihuahua, Coahuila, and Sonora through the concurrent release of sterile males and their parasitoid *Diachasmimorpha longicaudata* Ashmead (Hymenoptera: Braconidae)^81^. An early example assessed the suppression of *Ceratitis capitata* (Diptera: Tephritidae) within a 13 km^2^ area on the island of Maui, Hawaii. Concurrent releases of sterile males and the parasitoid *Diachasmimorpha tryoni* Cameron (Hymenoptera: Braconidae) stimulated a significantly increased parasitism rate and decreased pest density compared with a control site^82^. Other studies have confirmed the efficacy of combining SIT with biological control through field cage assays using *Cydia pomonella* Linnaeus (Lepidoptera: Tortricidae) and *Thaumatotibia leucotreta* Meyrick (Lepidoptera: Tortricidae)^57,83–85^. Given that these studies relied on already present pest and parasitoid populations, they were not limited by stringent regulations surrounding the importation and release of natural enemies^6^. The scene was therefore set to explore whether the release of sterile parasitoids could enable their utilisation to improve an eradication attempt against an unfolding pest incursion, which is pertinent as the feasibility of eradication is often greatest at low target pest densities^12,13^.

Although we have used *T. basalis* and its host *N. viridula* to investigate the KWT, this predominantly promotes the potential for implementing the KWT on the globally emerging pest *H. halys* with its parasitoid *T. japonicus*. The former is not yet established in the Southern Hemisphere, with the exception of Chile^86^, and represents a serious biosecurity threat owing to its highly polyphagous feeding habits^87^. For example, in New Zealand, *H. halys* has been repeatedly intercepted at the border in recent years^88,89^. The Environmental Protection Authority has granted permission for the introduction of *T. japonicus* into New Zealand, and its release from containment to support an eradication response against *H. halys*^90,91^. Additionally, Welsh et al.^24^ ascertained the irradiation biology for male *H. halys*, showing that a gamma-irradiation dose of 16 Gy induced 97% egg sterility after mating with fertile females. This pest-parasitoid system therefore represents an appropriate case study to investigate the viability of the KWT as an eradication response, and the irradiation biology of *T. japonicus* should be investigated now that feasibility is confirmed for *T. basalis*, though field efficacy trials must first be completed. Overall, this study has demonstrated the potential for this novel concept to be developed further. However, the complicated set of scientific, economic, and socio-political limitations to the initiation of eradication programmes, including the need for early detection and preparedness of tools^49,50^, may limit the development and implementation of novel approaches such as the KWT.

It is pertinent to note that for pest management, the release of sterile parasitoid wasps would only be applicable to an eradication response. Once a pest has established and spread beyond the possibility of eradication^13^, self-sustaining parasitoid release would be optimal to provide inexpensive, long-term classical biological control^1^.

## Conclusions and implications

This is the first experimental study to investigate the potential application of sterile CBC agent release. The successful permanent sterilisation of *T. basalis*, which retains its ability to kill host eggs, promotes further investigation into whether sterile parasitoids could be combined with SIT to improve eradication outcomes, with negligible risk of incurring irreversible non-target impacts after pest elimination. This could mitigate the need for extensive pre-release testing of parasitoids, which largely excludes them from eradication programmes owing to the need to act quickly whilst pest densities remain low. Further research required for the development of the KWT includes systematic fitness assessments of sterile parasitoids, population modelling, and efficacy trials for the KWT scenario, which would involve laboratory and field cage trials, as well as open field trials.

## Data Availability

The datasets generated and/or analysed during the current study are uploaded to Researchgate ([link to URL when uploaded]), and are available from the corresponding author on reasonable request.
